# The issue of atherosclerosis in primary glomerulonephritis

**DOI:** 10.1590/2175-8239-JBN-2023-E015en

**Published:** 2023-12-08

**Authors:** Maurilo Leite, Alinie Pichone

**Affiliations:** 1 Universidade Federal do Rio de Janeiro, Hospital Universitário Clementino Fraga Filho, Rio de Janeiro, RJ, Brazil.

Patients with glomerular disease and proteinuria may exhibit increased risk for cardiovascular disease, in part due to traditional risk factors such as associated hypertension, dyslipidemia, age, gender, and occasional obesity. In addition, it has been well documented that more advanced stages of chronic kidney disease (CKD) that may occur in cases of primary glomerulonephritis (PG), are associated with several nontraditional risk factors. The list includes anemia, retention of sodium and uremic toxins, chronic inflammation, increased oxidative stress, and mineral bone disorder (MBD)^1,2^.

Assessment of possible explanations for the high prevalence of atherosclerosis in PG patients is crucial for therapeutic and preventive strategies. In the present issue of BJN, Hageman and coworkers^3^ initially aimed to investigate the role of MBD on atherogenesis in a group of PG patients. These patients exhibited eGFR that ranged from 48 to 105.12 mL/min/1.73m^2^ with mean GFR of 80.3 mL/min/1.73m^2^ per minute. In fact, it is conceivable that CKD patients from stage 2 onward, which notably exhibit increasing serum levels of FGF-23 and decreasing levels of α-Klotho, might develop left ventricular hypertrophy and become at increased risk of cardiovascular events, including vascular calcification^2,4^. The group of PG patients recruited by Hageman and coworkers was compared to a control group of volunteers. The results showed differences between groups in the percentage of hypertensive individuals and statins use, but not in dyslipidemia, eGFR, and carotid intima-media thickness (CIMT). Despite CIMT values below those known to be associated with atherosclerosis, these results can be comprehensively interpreted as evidence for early atherogenesis in the PG group. They also assessed flow-mediated vasodilation (FMV), and both CIMT and FMV were not correlated with the levels of FGF-23 in the PG patients. Nevertheless, both CIMT and FMV were correlated with eGFR levels, as previously shown by Kastarinen et al.^5^ and Yilmaz et al.^6^.

The role that early MBD can play in the development of cardiovascular disease in PG patients is the subject of investigation, and the key points to be addressed in stage 2 and 3 CKD patients should always be linked to early changes in serum levels of FGF-23 and α-Klotho. Moreover, PG is often linked to hypertension and systemic inflammation in addition to proteinuria, nephrotic dyslipidemia, and the risk of thrombosis, regardless of GFR. In fact, the study by Mackinnon et al.^7^ found abnormalities in endothelial function in a population of PG patients with well-preserved renal function compared to controls and adjusted for mean arterial pressure and BMI. Of note, systemic inflammation was more pronounced in the PG group, as assessed by sensitive CRP.

As part of the conclusion, the study by Hageman et al.^3^ found a significant inverse correlation between FMV and serum uric acid in the PG group, although information regarding serum uric acid levels in PG and control groups are lacking. The mechanisms of hyperuricemia-mediated endothelial dysfunction were recently reviewed by Wei et al.^8^ and include decreased nitric oxide production, oxidative stress, epithelial-to-mesenchymal transition, endothelial cell insulin resistance, and activation to release inflammatory cytokines. However, the precise mechanisms underlying possible impaired uric acid excretion in the setting of specific glomerular diseases needs to be fully explored.

The authors also emphasize the correlation between systolic blood pressure and CIMT, which is noteworthy. Hypertension is somewhat to be expected in PG, and the mechanisms associated are well discussed in a review by Ihm^9^. Increased arterial media thickness and endothelial damage may be histopathological consequences of hypertension and lead to arteriosclerosis.

Overall, the most important message from the paper by Hageman and coworkers^3^ comes from the analysis between the two groups of patients. The PG group had higher percentage of hypertensive individuals as well as lower levels of eGFR compared to control volunteers. These results could, at least in part, explain the different values of CIMT, a direct index for atherosclerosis. In fact, previous documentation by Hutton et al.^10^ in which CV risk of patients with CKD and glomerulonephritis did not differ from other CKD patients from other causes, suggesting that glomerulonephritis itself does not add risk but the level of renal function does. Accordingly, in the study by Hageman et al.^3^, both FMV, a marker of endothelial function, and CIMT were correlated with eGFR in the PG group.

Early changes in mineral bone homeostasis in stages 2 and 3, as detected by the increase in serum FGF-23, were not correlated with CIMT and FMV in this group of PG patients. However, measurement of serum α-Klotho might be crucial for a throughout evaluation of CV risk factors in patients with primary glomerulonephritis in early CKD stages.
